# Species Boundaries and Host Range of Tortoise Mites (Uropodoidea) Phoretic on Bark Beetles (Scolytinae), Using Morphometric and Molecular Markers

**DOI:** 10.1371/journal.pone.0047243

**Published:** 2012-10-11

**Authors:** Wayne Knee, Frédéric Beaulieu, Jeffrey H. Skevington, Scott Kelso, Anthony I. Cognato, Mark R. Forbes

**Affiliations:** 1 Department of Biology, Carleton University, Ottawa, Ontario, Canada; 2 Canadian National Collection of Insects, Arachnids and Nematodes, Agriculture and Agri-Food Canada, Ottawa, Ontario, Canada; 3 Department of Entomology, Michigan State University, East Lansing, Michigan, United States of America; Roehampton University, United Kingdom

## Abstract

Understanding the ecology and evolutionary history of symbionts and their hosts requires accurate taxonomic knowledge, including clear species boundaries and phylogenies. Tortoise mites (Mesostigmata: Uropodoidea) are among the most diverse arthropod associates of bark beetles (Curculionidae: Scolytinae), but their taxonomy and host associations are largely unstudied. We tested the hypotheses that (1) morphologically defined species are supported by molecular data, and that (2) bark beetle uropodoids with a broad host range comprise cryptic species. To do so, we assessed the species boundaries of uropodoid mites collected from 51 host species, across 11 countries and 103 sites, using morphometric data as well as partial cytochrome oxidase I (COI) and nuclear large subunit ribosomal DNA (28S). Overall, morphologically defined species were confirmed by molecular datasets, with a few exceptions. Twenty-nine of the 36 uropodoid species (*Trichouropoda*, *Nenteria* and *Uroobovella*) collected in this study had narrow host ranges, while seven species had putative broad host ranges. In all but one species, *U. orri*, our data supported the existence of these host generalists, which contrasts with the typical finding that widespread generalists are actually complexes of cryptic specialists.

## Introduction

Increased access to nucleotide sequencing over the last twenty years has led to exponential growth of molecular-based taxonomy [1]. Modern molecular techniques provide powerful tools to assess species boundaries, and cryptic species (species distinguishable by no or overlooked subtle morphological differences) are being discovered increasingly in a wide range of invertebrate groups [2]–[4]. Species boundaries of symbionts are frequently assessed using molecular markers, and it is often revealed that an apparent widespread host generalist is not a generalist, but rather a complex of cryptic species with narrower host ranges. For instance, *Ixodes uriae* (Ixodidae) was previously considered to be a host generalist, but microsatellite analysis showed strong genetic divergence across host species, suggesting that *I. uriae* represents multiple host races with relatively narrower host ranges [5], [6]. Morphological and molecular analyses of *Uroobovella nova* (Urodinychidae), a single widespread putative generalist uropodoid species collected from silphid beetles worldwide, is actually a complex of cryptic species with varying degrees of host specificity [7].

Bark beetles (Curculionidae: Scolytinae) are a prominent group of wood-borers that feed and mate in the cambium or xylem of numerous tree species worldwide [8]. Mites are one of the most common and diverse associates of scolytines. For instance, 97 species of mites representing 65 genera and 40 families have been collected from under the bark of scolytine infested pine trees [9]. Many or most of these mites reside, feed and reproduce in the galleries of bark beetles, and they attach to dispersing scolytines, hitching a ride to new host trees or coarse woody debris, which would otherwise be difficult to access for most free-living mites.

Uropodoids (Acari: Mesostigmata), or tortoise mites, are among the most frequently collected mite associates of bark beetles, and include three genera *Trichouropoda, Nenteria* (Trematuridae) and *Uroobovella* (Urodinychidae). Scolytine-associated uropodoids are often found at a relatively high prevalence (e.g. up to 36% of 8475 beetles had mites in Louisiana; [10]. The superfamily Uropodoidea is represented by over 2,000 described species worldwide, many of which occur in patchy habitats such as nests, woody debris, and dung [11]. Phoresy is therefore a prerequisite for dispersal between such patchy habitats, and deutonymphal uropodoids glue themselves to their host with an anally secreted pedicel. The feeding habits of uropodoids are poorly known but typically they are considered to be omnivorous, feeding on fungal hyphae, slow moving prey, or small particulate matter [12]. The deutonymphs of some species associated with scolytines have been reported as feeding on nematodes and or fungi [13], [14], as well as the eggs and larvae of their bark beetle hosts [15], [16].

Many acarological studies have used mitochondrial cytochrome oxidase I (COI) and nuclear large subunit ribosomal DNA (28S), either alone or combined with other markers, to elucidate species boundaries, uncover cryptic species, and assess phylogenetic relationships of mites [17]–[22]. In this study, we employed morphological and molecular markers (COI and 28S D2–D4) to explore the species boundaries of bark beetle-associated uropodoids and to assess whether morphological species concepts are supported by molecular data. Additionally, we tested whether generalists are truly single species with broad host preferences or instead complexes of cryptic species with narrower host ranges, using quantitative morphological and molecular analyses.

## Materials and Methods

### Biological Material

Bark beetle specimens were collected across 11 countries and 103 sites, with the majority of sites in Canada and the USA. Canadian specimens were collected in Ontario by W.K. and in various provinces by the Canadian Food Inspection Agency (CFIA) staff as part of the Invasive Alien Species Monitoring program, and examined by W.K. with permission. Specimens from the USA and other countries were collected by A.I.C., and examined by W.K. with permission. All necessary permits and permissions were obtained for the described field studies. Field studies were conducted with a permit to collect in Ontario Provincial Parks issued by Ontario Parks and coordinated by B. Steinberg and B. Crins, as well as permission from private landowners to sample on their property.

In Ontario, bark beetles were collected from mid-April to early August 2009 across four study sites: Algonquin Provincial Park site 1 (45.902, −77.605), Algonquin PP site 2 (45.895, −78.071), one site near Pakenham (45.33, −76.371), and another on Hwy 132 near Dacre (45.369, −76.988). Four Lindgren traps with propylene glycol were placed in each study site. Traps were baited with 95% ethanol and/or α-pinene lures (Synergy Semiochemicals). Traps were emptied every two weeks, trap lures were replaced every eight weeks, and the propylene glycol insecticide was replaced at each visit. Bark beetles were placed individually into 1.5 ml microfuge tubes with 95% ethanol and stored at −20°C. Scolytines were identified to species using keys [8], [23], and tribes were based on the literature [24]. Beetles were examined for uropodoid mites using a dissecting microscope, and all mites found were removed and placed into a 0.5 ml microfuge tube with 95% ethanol and stored at −20°C.

A portion of the bark beetles collected by CFIA staff in 2009 from Canadian provinces, as well as scolytine specimens collected by A.I.C. from USA and several other countries were examined by W.K. for uropodoid mites, and all mites found were removed and stored in 95% ethanol at −80°C. Four species of uropodoids (*Uroobovella* spp. 1–4) collected from *Nicrophorus* beetles (Silphidae) in Ontario were used as outgroup specimens. Although the outgroup species are in the same genus as some of the ingroup, the generic position of the outgroup species is contentious, and they are associated with a different family of beetles. Following DNA extraction, mites were recovered from the extraction buffer and slide-mounted in a polyvinyl alcohol medium, and slides were cured on a slide warmer at about 40°C for 3–4 days. Slide-mounted specimens were examined using a compound microscope (Leica DM 5500B or Nikon 80I) and identified to species (or morphospecies) using taxonomically informative morphological characters based on species descriptions from the literature [25]–[30]. Species were identified prior to examining the molecular reconstructions, and in any instances where a conflicting result emerged between the molecular data and morphology-based identifications, both datasets were reexamined. Voucher specimens are deposited in the Canadian National Collection of Insects, Arachnids and Nematodes, in Ottawa, Canada, and the Michigan State University A.J. Cook Arthropod Research Collection, East Lansing, USA.

### DNA Extraction, Amplification and Sequencing

Total genomic DNA was extracted from whole specimens for 24 hours using a DNeasy Tissue kit (Qiagen Inc., Santa Clara, CA, USA). Following extraction, mites were removed from the extraction buffer, and genomic DNA was purified following the DNeasy Tissue kit protocol.

PCR amplifications were performed in a total volume of 25 µl, with 13 µl ddH_2_O, 2.5 µl 10× PCR buffer, 2.5 µl 25 mM MgCl_2_, 0.5 µl of each 10 µM primer, 0.5 µl 10 mM dNTPs, 0.5 µl Taq DNA polymerase (Promega Corp., Madison, WI, USA), and 5 µl genomic DNA template. In the instances where semi-nested or nested primers were employed, 1 µl of primary PCR product was used as template and the ddH_2_0 was increased to 17 µl. PCR amplification cycles were performed on an Eppendorf ep Gradient S Mastercycler (Eppendorf AG, Hamburg, Germany). Primer pairs LCO1490+ LoDog, and LCO1490+ BB R4 (Table 1), were used to amplify 643 and 603 bp fragments, respectively, of the mitochondrial COI gene. Specimens that did not produce detectable PCR products using either of these primer pairs were reamplified using 1 µl of the primary PCR product and semi-nested, LCO1490+ BB R3Lo, or nested, BB F + BB R3Lo, primer combinations (Table 1), which amplified 592 and 475 bp fragments, respectively. The thermocycler protocol for COI amplification was as follows: initial denaturation cycle at 94°C for 3 min, followed by 40 cycles of 94°C for 45 s, primer annealing at 45°C for 45 s, 72°C for 1 min, and a final extension at 72°C for 5 min. The primer annealing temperature was reduced to 43°C when primer BB R4 was employed.

**Table 1:** Primer sequences (5′–3′) used to amplify partial COI and 28S D2–D4 sequences from uropodoid mites collected from bark beetles (*primers from this study).

Gene	Primer	Sequence 5′–3′	Reference
COI	LCO1490	GGTCAACAAATCATAAAGATATTGG	51
	BB F	TAATTGGWRATGAYCAAATTTTTAA	*
	BB R2	AATHGTDGTAATAAAATTAATTGA	*
	BB R3Lo	CCTCCTGCTAADACHGG	*
	BB R4	GTATAGTAATRGCTCCTGC	*
	LoDog	GGRTCAAAAAAAGAWGTRTTRAARTTTCG	*
28S	D23F	GAGAGTTCAAGAGTACGTG	52
	28S Fb	GAGTACGTGAAACCGCWTWGA	*
	28Sa	GACCCGTCTTGAAACACGG	53 (modified)
	28S F1	GGCGHAATGAAATGTGAAGG	*
	28S R3	GGCTTCRTCTTGCCCAGGC	*
	28S R4	GGCTTCGTCTTGCCCAGGC	*
	28Sb	CGGAAGGAACCAGCTAC	53 (modified)
	28S R2	CCAGTTCTGCTTACCAAAAATGG	*

**Figure 1:**
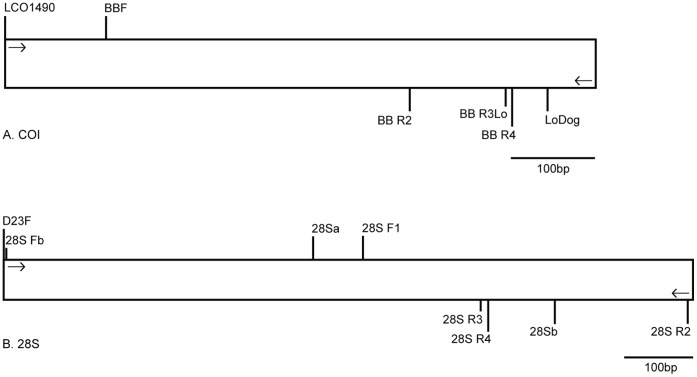
Primer map showing the relative location of primers used to amplify. (A) partial COI, and (B) 28S D2–D4 sequences from uropodoid mites collected from bark beetles.

Primer pairs D23F +28S R2, and 28S Fb +28S R2 (Table 1), were used to amplify a 990 and 980 bp fragment, respectively, from the 5′ end of the nuclear ribosomal 28S gene, spanning the D2–D4 region. In the instances where neither primer pair produced a detectable PCR product, the specimens were reamplified using 1 µl of the primary PCR product and semi-nested primer pairs, D23F +28Sb or 28S Fb +28Sb, which amplified an 800 and 790 bp fragment of 28S rDNA, respectively (Table 1). The PCR protocol for D23F +28S R2, and D23F +28Sb was as follows: initial denaturation cycle at 95°C for 2 min, followed by 30 cycles of 95°C for 1 min, primer annealing at 44°C for 1.5 min, 72°C for 2 min, and a final extension at 72°C for 10 min. The primer annealing temperature was changed to 56°C for 28S Fb +28S R2, and it was changed to 50°C for 28S Fb +28S R2. Additional primers were designed to amplify COI and 28S from uropodoids; all primers designed or used in this study are shown in the primer map (Table 1, Fig. 1).

Amplified products and negative controls were visualized on 1% agarose electrophoresis gels, and purified using pre-cast E-Gel CloneWell 0.8% SYBR Safe agarose gels (Invitrogen, Carlsbad, CA, USA) following the protocol of [31]. Sequencing reactions were performed in a total reaction volume of 10 µl, with 3 µl ddH_2_O, 1.5 µl of 5× sequencing buffer, 0.5 µl of primer, 1 µl of BigDye Terminator (PE Applied Biosystems, Foster City, CA, USA), and 4 µl of purified PCR product. Sequencing was performed at the Agriculture & Agri-Food Canada, Eastern Cereal and Oilseed Research Centre Core Sequencing Facility (Ottawa, ON, Canada). Purification of sequencing reactions was performed using the ABI ethanol/EDTA/sodium acetate precipitation protocol and reactions were analysed on an ABI 3130×l Genetic Analyzer (PE Applied Biosystems, Foster City, CA, USA).

### Sequence Alignment and Phylogenetic Analysis

Sequence chromatograms were edited and contiguous sequences were assembled using Sequencher v4.7 (Gene Codes Corp., Ann Arbor, MI, USA). COI sequences were aligned manually in Mesquite v2.74 [32] according to the translated amino acid sequence. 28S was initially aligned in ClustalX v2.0.12 [33] with the default settings, and subsequently adjusted manually in Mesquite, no regions were excised, and due to the absence of any secondary structure for mites for this gene region, no secondary structure alignment was performed. Sequences have been submitted to GenBank (Table 2).

**Table 2:** Collection locations and host species records of uropodoid mites collected from scolytines (ingroup) and *Nicrophorus* beetles (outgroup) with GenBank accession no. for COI and 28S (*Uroob = *Uroobovella, Trich* = *Trichouropoda, Nent* = *Nenteria*).

**Beetle no.**	**Beetle species**	**Collection location**	**Lat**	**Long**	**Date**	Mite species*	**COI**	**28S**
1 - WKB4051	*Pityokteines sparsus*	Can, ON, Hwy 132, Dacre	45.369	−76.988	16 v 2009	*Uroob. orri*	JN992226	–
2 - WKB4057	*Orthotomicus caelatus*	Can, ON, Algonquin P.P. 1	45.902	−77.605	16 v 2009	*Uroob.* n.sp. 6	JN992227	–
3 - WKB4095	*Gnathotrichus materiarius*	Can, ON, Algonquin P.P. 2	45.895	−78.071	16 v 2009	*Trich. parisiana*	JN992184	–
4 - WKB4109	*Ips grandicollis*	Can, ON, Algonquin P.P. 2	45.895	−78.071	16 v 2009	*Trich. australis*	–	–
5 - WKB4190	*Pityokteines sparsus*	Can, ON, Algonquin P.P. 2	45.895	−78.071	28 v 2009	*Trich. moseri*	JN992171	–
6 - WKB4232	*Polygraphus rufipennis*	Can, ON, Carbine Rd.	45.330	−76.371	16 v 2009	*Uroob. orri*	–	–
7 - WKB4429	*Dendroctonus valens*	Can, ON, Algonquin P.P. 2	45.895	−78.071	16 v 2009	*Uroob. americana*	JN992202	–
8 - WKB4850	*Polygraphus rufipennis*	Can, AB, Fort McMurray	56.016	−110.88	23 vii 2009	*Trich. moseri*	JN992172	–
9 - WKB4869	*Dryocoetes affaber*	Can, AB, Fort McMurray	56.016	−110.88	29 vi 2009	*Uroob. orri*	–	–
10 - WKB4943	*Hylesinus aculeatus*	Can, ON, Hwy 132, Dacre	45.369	−76.988	1 v 2009	*Trich. bipilis*	JN992155	–
11 - WKB4987	*Ips pini*	Can, ON, Algonquin P.P. 1	45.902	−77.605	1 v 2009	*Trich. australis*	JN992139	–
12 - WKB4995	*Trypodendron retusum*	Can, ON, Algonquin P.P. 1	45.902	−77.605	1 v 2009	*Trich. parisiana*	–	–
13 - WKB5224	*Polygraphus rufipennis*	Can, ON, Algonquin P.P. 1	45.902	−77.605	28 v 2009	*Uroob. orri*	JN992228	–
14 - WKB5226	*Dryocoetes affaber*	Can, ON, Algonquin P.P. 1	45.902	−77.605	28 v 2009	*Uroob. orri*	JN992229	–
15 - WKB5261	*Hylastes porculus*	Can, ON, Algonquin P.P. 1	45.902	−77.605	28 v 2009	*Uroob. dryocoetes*	JN992211	–
16 - WKB5344	*Gnathotrichus materiarius*	Can, ON, Algonquin P.P. 1	45.902	−77.605	28 v 2009	*Trich. parisiana*	JN992185	–
17 - WKB5351	*Dendroctonus valens*	Can, ON, Algonquin P.P. 1	45.902	−77.605	28 v 2009	*Uroob. dryocoetes*	–	–
18 - WKB5563	*Pityogenes hopkinsi*	Can, ON, Algonquin P.P. 2	45.895	−78.071	28 v 2009	*Trich.* n.sp. 3	–	–
19 - WKB5564	*Polygraphus rufipennis*	Can, ON, Algonquin P.P. 2	45.895	−78.071	28 v 2009	*Trich. moseri*	–	–
20 - WKB5568	*Ips pini*	Can, ON, Algonquin P.P. 2	45.895	−78.071	28 v 2009	*Trich. australis*	–	–
21 - WKB5639	*Orthotomicus caelatus*	Can, ON, Algonquin P.P. 2	45.895	−78.071	28 v 2009	*Uroob.* n.sp. 6	JN992230	–
22 - WKB5682	*Dryocoetes autographus*	Can, ON, Algonquin P.P. 1	45.902	−77.605	25 vi 2009	*Uroob. dryocoetes*	JN992212	–
23 - WKB5759	*Ips grandicollis*	Can, ON, Algonquin P.P. 1	45.902	−77.605	25 vi 2009	*Trich. lamellosa*	–	–
24 - WKB5759	*Ips grandicollis*	Can, ON, Algonquin P.P. 1	45.902	−77.605	25 vi 2009	*Uroob. orri*	JN992231	–
25 - WKB5797	*Hylurgops pinifex*	Can, ON, Algonquin P.P. 2	45.895	−78.071	25 vi 2009	*Trich. hirsuta*	–	–
26 - WKB5882	*Hylastes porculus*	Can, ON, Algonquin P.P. 2	45.895	−78.071	25 vi 2009	*Trich. hirsuta*	JN992167	JN992260
27 - WKB5970	*Dendroctonus ponderosae*	Can, AB, Grande Prairie			2007	*Trich. lamellosa*	JN992170	JN992261
28 - WKHD001	*Gnathotrichus materiarius*	Can, QC, La Patrie, Route 212	46.345	−72.576	22 v 2009	*Trich. parisiana*	–	–
29 - WKHD004	*Pityokteines sparsus*	Can, QC, La Patrie, Route 212	46.345	−72.576	22 v 2009	*Uroob. orri*	JN992232	–
30 - WKHD008	*Dendroctonus valens*	Can, QC, La Patrie, Route 212	46.345	−72.576	22 v 2009	*Uroob. americana*	–	–
31 - WKHD009	*Polygraphus rufipennis*	Can, QC, East Hereford	45.029	−71.505	22 v 2009	*Uroob. orri*	–	–
32 - WKHD010	*Gnathotrichus materiarius*	Can, QC, East Hereford	45.029	−71.505	22 v 2009	*Trich. parisiana*	–	–
34 - WKHD012	*Gnathotrichus materiarius*	Can, QC, Pont Rouge	46.806	−71.679	05 vi 2009	*Trich. parisiana*	JN992186	–
35 - WKHD014	*Polygraphus rufipennis*	Can, QC, Pont Rouge	46.806	−71.679	05 vi 2009	*Uroob. dryocoetes*	JN992213	–
36 - WKHD018	*Dendroctonus rufipennis*	Can, NS, West Northfield			01 vi 2009	*Uroob. orri*	–	–
37 - WKHD030	*Hylastes porculus*	Can, NS, Westfield	44.403	−64.975	28 v 2009	*Uroob. dryocoetes*	JN992214	–
38 - WKHD037	*Hylastes porculus*	Can, NB, Bayside, Route 127	45.205	−67.140	15 vi 2009	*Uroob. dryocoetes*	–	–
39 - WKHD042	*Xyleborinus saxesenii*	Can, BC, Stanley Park, Pipeline Dr.			06 vi 2008	*Trich. parisiana*	JN992187	–
40 - WKHD057	*Gnathotrichus materiarius*	Can, QC, Parc des iles de Boucherville	45.601	−73.466	26 v 2009	*Trich. parisiana*	–	–
41 - WKHD062	*Dendroctonus valens*	Can, QC, Sorel-Tracy	46.030	−73.083	09 vi 2009	*Uroob. americana*	JN992203	–
42 - WKHD065	*Gnathotrichus materiarius*	Can, QC, Sorel-Tracy	46.030	−73.083	09 vi 2009	*Uroob. orri*	–	–
43 - WKHD066	*Hylastes porculus*	Can, QC, Sorel-Tracy	46.030	−73.083	09 vi 2009	*Uroob. dryocoetes*	JN992215	JN992277
44 - WKHD067	*Dryocoetes autographus*	Can, QC, Sorel-Tracy	46.030	−73.083	09 vi 2009	*Uroob. dryocoetes*	–	–
45 - WKHD070	*Dryocoetes affaber*	Can, QC, Sorel-Tracy	46.030	−73.083	09 vi 2009	*Uroob. dryocoetes*	–	–
46 - WKHD075	*Hylastes ruber*	Can, BC, McPhee Creek Rd.	49.323	−117.61	29 iv 2009	*Trich. fallax*	JN992166	JN992259
47 - WKHD078	*Hylurgops pinifex*	Can, NS, Greenfield	44.335	−64.915	11 vi 2009	*Trich. fallax*	–	–
48 - WKHD079	*Dendroctonus rufipennis*	Can, NS, Annapolis, Granville ferry	44.810	−65.537	22 vi 2009	*Trich. alascae*	JN992137	–
49 - WKHD079	*Dendroctonus rufipennis*	Can, NS, Annapolis, Granville ferry	44.810	−65.537	22 vi 2009	*Uroob. orri*	JN992233	–
50 - WKHD080	*Dendroctonus rufipennis*	Can, NS, Victoria Beach	44.703	−65.747	22 vi 2009	*Uroob. orri*	JN992234	–
51 - WKHD085	*Dendroctonus rufipennis*	Can, NS, Blomidon, Stewart Mtn. Rd.	45.227	−64.397	19 vi 2009	*Trich. alascae*	JN992138	JN992253
52 - WKHD085	*Dendroctonus rufipennis*	Can, NS, Blomidon, Stewart Mtn. Rd.	45.227	−64.397	19 vi 2009	*Uroob. orri*	JN992235	–
53 - WKHD114	*Dendroctonus rufipennis*	Can, QC, Degelis	47.561	−68.644	16 vi 2009	*Uroob. orri*	–	–
54 - WKHD116	*Hylastes porculus*	Can, QC, Saint Come De liniere	46.014	−70.483	23 vi 2009	*Uroob. dryocoetes*	JN992216	–
55 - WKHD117	*Gnathotrichus materiarius*	Can, QC, Degelis	47.551	−68.642	26 vi 2009	*Trich. parisiana*	–	–
56 - WKHD118	*Hylastes porculus*	Can, QC, Degelis	47.551	−68.642	26 vi 2009	*Uroob. dryocoetes*	JN992217	–
57 - WKHD120	*Dendroctonus valens*	Can, QC, Pont Rouge	46.562	−71.545	08 vi 2009	*Uroob. americana*	JN992204	–
58 - WKHD121	*Dendroctonus valens*	Can, QC, Saint Pamphile	46.943	−69.764	17 vi 2009	*Uroob. dryocoetes*	JN992218	–
59 - WKHD129	*Dendroctonus rufipennis*	Can, QC, Saint Pamphile	46.947	−69.761	17 vi 2009	*Uroob. orri*	–	–
60 - WKHD130	*Dryocoetes autographus*	Can, NB, Monument	45.954	−67.767	24 vi 2009	*Uroob. dryocoetes*	JN992219	–
61 - WKHD133	*Dendroctonus rufipennis*	Can, NS, Sheet Harbour	44.907	−62.491	19 vi 2009	*Uroob. orri*	JN992236	–
62 - WKHD136	*Polygraphus rufipennis*	Can, NS, Sheet Harbour	44.907	−62.491	19 vi 2009	*Uroob. orri*	JN992237	–
63 - WKHD140	*Dryocoetes autographus*	Can, NS, Sheet Harbour	44.909	−62.503	19 vi 2009	*Uroob. dryocoetes*	JN992220	–
64 - WKHD142	*Dryocoetes affaber*	Can, NS, Sheet Harbour	44.909	−62.503	19 vi 2009	*Trich. hirsuta*	–	–
65 - WKHD142	*Dryocoetes affaber*	Can, NS, Sheet Harbour	44.909	−62.503	19 vi 2009	*Uroob. orri*	JN992238	–
66 - WKHD149	*Polygraphus rufipennis*	Can, QC, Cookshire	45.389	−71.513	02 vii 2009	*Trich. hirsuta*	–	–
67 - WKHD158	*Dryocoetes autographus*	Can, QC, Cookshire	45.389	−71.513	02 vii 2009	*Uroob. dryocoetes*	JN992221	–
68 - WKHD169	*Dryocoetes affaber*	Can, QC, Cookshire	45.389	−71.513	02 vii 2009	*Uroob. dryocoetes*	JN992222	–
69 - WKHD172	*Dryocoetes affaber*	Can, QC, Saint Malo	45.197	−71.527	02 vii 2009	*Uroob. dryocoetes*	–	–
70 - WKHD175	*Hylastes porculus*	Can, QC, La Patrie, Route 212	46.345	−72.576	02 vii 2009	*Uroob. dryocoetes*	JN992223	–
71 - WKHD177	*Dryocoetes autographus*	Can, QC, La Patrie, Route 212	46.345	−72.576	02 vii 2009	*Uroob. dryocoetes*	–	–
72 - WKHD178	*Orthotomicus caelatus*	Can, NS, Goodwood	44.603	−63.677	27 v 2009	*Uroob.* n.sp. 6	JN992239	JN992278
73 - WKHD179	*Ips pini*	Can, NS, Goodwood	44.603	−63.677	27 v 2009	*Trich. australis*	JN992140	JN992254
74 - WKHD181	*Polygraphus rufipennis*	Can, NS, Purcell’s Cove	44.624	−63.575	03 vi 2009	*Uroob. orri*	JN992240	–
75 - WKHD182	*Dryocoetes affaber*	Can, NS, Purcell’s Cove	44.624	−63.575	03 vi 2009	*Uroob. orri*	JN992241	–
76 - WKHD183	*Dendroctonus rufipennis*	Can, NS, Purcell’s Cove	44.624	−63.575	13 vii 2009	*Uroob. orri*	–	–
77 - WKHD184	*Gnathotrichus materiarius*	Can, NS, Debert, Industrial Park	45.428	−63.429	25 vi 2009	*Trich. parisiana*	JN992188	–
78 - WKHD185	*Ips pini*	Can, NS, Debert, Industrial Park	45.428	−63.429	25 vi 2009	*Trich. australis*	JN992141	–
79 - WKHD189	*Ips borealis*	Can, NS, Debert, Industrial Park	45.428	−63.429	25 vi 2009	*Trich. polytricha*	JN992191	–
80 - WKHD193	*Dryocoetes autographus*	Can, NS, Debert, Industrial Park	45.428	−63.429	25 vi 2009	*Uroob. dryocoetes*	JN992224	–
81 - WKHD194	*Dryocoetes affaber*	Can, QC, Saint Roch de Mekinac	46.792	−72.748	23 vi 2009	*Uroob. orri*	–	–
82 - WKHD199	*Hylastes porculus*	Can, QC, Saint Severin, Route 159	46.686	−72.525	23 vi 2009	*Uroob. dryocoetes*	JN992225	–
83 - WKHD204	*Ips grandicollis*	Can, ON, Brampton	43.708	−79.728	06 vii 2009	*Trich. australis*	JN992142	–
84 - WKHD208	*Ips grandicollis*	Can, ON, Argentia Rd. Century Ave	43.598	−79.744	07 vii 2009	*Trich. australis*	JN992143	JN992255
85 - WKHD228	*Ips pini*	Can, QC, Boucherville	45.601	−73.466	09 vii 2009	*Trich. australis*	JN992144	–
86 - WKHD230	*Ips pini*	Can, ON, Argentia Rd. Century Ave	43.598	−79.744	20 vii 2009	*Trich. australis*	–	–
87 - WKHD232	*Ips grandicollis*	Can, ON, New Market, 500 Water St.	44.047	−79.456	23 vii 2009	*Uroob. orri*	JN992242	–
88 - WKHD234	*Ips pini*	Can, ON, New Market, 500 Water St.	44.047	−79.456	23 vii 2009	*Trich. australis*	JN992145	–
89 - WKHD235	*Polygraphus rufipennis*	Can, QC, Saint Zacharie	46.130	−70.262	21 vii 2009	*Trich. moseri*	JN992173	JN992262
90 - WKHD236	*Polygraphus rufipennis*	Can, QC, Woburn	45.342	−70.898	21 vii 2009	*Trich. moseri*	JN992174	–
91 - WKHD237	*Polygraphus rufipennis*	Can, QC, Saint Benjamin	46.268	−70.617	21 vii 2009	*Trich. moseri*	–	–
92 - WKHD252	*Ips borealis*	Can, NS, Hantsport, Cobesquid Bay	45.099	−64.184	21 vii 2009	*Trich. polytricha*		–
93 - WKHD254	*Ips pini*	Can, NS, Hantsport, Cobesquid Bay	45.099	−64.184	11 viii 2009	*Trich. australis*	JN992146	–
94 - WKHD261	*Hylastes subopacus*	USA, NM, Bernalillo			10 x 2008	*Nent. chiapasa*	–	–
95 - WKB5929	*Dendroctonus valens*	Can, ON, Algonquin P.P. 2	45.895	−78.071	25 vi 2009	*Uroob. americana*	JN992205	–
96 - WKB5639	*Orthotomicus caelatus*	Can, ON, Algonquin P.P. 2	45.895	−78.071	28 v 2009	*Uroob.* n.sp. 6	JN992243	JN992279
97 - WKB5929	*Dendroctonus valens*	Can, ON, Algonquin P.P. 2	45.895	−78.071	25 vi 2009	*Uroob. americana*	JN992206	JN992275
98 - MSU001	*Pityophthorus* sp.	USA, CA, El Dorado N.F. Ice House Res.	38.5	−120.22	25 v 2007	*Trich.* n.sp. 2	JN992178	JN992265
99 - MSU004	*Dendroctonus valens*	USA, OH, Secrest Arboretum	40.782	−81.916	v 2007	*Uroob. americana*	–	–
100 - MSU006	*Ficicis* sp.	China, Yunnan, Xishuangbanna	22.163	100.871	30 v 2008	*Uroob. australiensis*	JN992210	–
101 - MSU010	*Dendroctonus valens*	USA, PA, Keystone Rd.	40.739	−76.308	30 iv 2009	*Uroob. americana*	–	–
102 - MSU012	*Polygraphus* sp.	Thailand, Doi Pui			iv 2005	*Trich. polygraphi*	–	–
103 - MSU014	*Scolytus ventralis*	USA, CA, El Dorado N.F. Ice House Res.	38.5	−120.22	17 vi 2003	*Trich.* n.sp. 10	JN992175	JN992263
104 - MSU016	*Hylurgops rugipennis pinifex*	USA, UT, Ashley N.F., Gray Head Peak	39.54	−110.45	11 vi 2003	*Trich. fallax*	–	–
105 - MSU020	*Monarthrum dentigerum*	USA, TX, Davis Mt. S.P.			25 v 2001	*Trich.* n.sp. 8	–	–
106 - MSU024	*Monarthrum dentigerum*	USA, TX, Big Bend N.P.			iv 2004	*Trich.* n.sp. 8	–	–
107 - MSU025	*Hylurgops* sp.	Mex, South of Amecameca	19.016	−98.741	11 v 2004	*Uroob. vinicolora*	JN992248	–
108 - MSU028	*Hylastes* sp.	USA, WI, Cobma			11 iv 2004	*Trich. perissopos*	–	–
109 - MSU030	*Dendroctonus valens*	USA, WI, nr. Madison			v 2005	*Uroob. americana*	JN992207	–
110 - MSU032	*Pseudips mexicanus*	Mex, Jalisco			5 xi 2003	*Nent. moseri*	JN992136	JN992252
111 - MSU036	*Pityokteines curvidens*	Croatia			2003	*Uroob. orri*	JN992244	JN992280
112 - MSU038	*Pseudips mexicanus*	Mex, Jalisco, nr. Ciudad Guzman			9 ii 2006	*Trich.* n.sp. 9	JN992181	–
113 - MSU040	*Orthotomicus erosus*	Italy, Tuscany, nr. San Gusme	43.360	11.501	29 xii 2006	*Trich.* n.sp. 4	JN992179	JN992266
114 - MSU045	*Ips hunteri*	USA, UT, Ashley N.F., Hwy 191	40.43	−109.29	10 vi 2003	*Trich. polytricha*	–	–
115 - MSU049	*Ips pilifrons utahensis*	USA, CO, San Isabel N.F. Monarch Pass	38.31	−106.19	9 vi 2003	*Trich. polytricha*	–	–
116 - MSU050	*Ips cribricollis*	USA, NM, Big Burro Mts			20 viii 2003	*Trich. australis*	JN992147	–
117 - MSU051	*Ips perturbatus*	USA, MN, Cascade River Park			12 vi 2001	*Trich. polytricha*	JN992192	–
118 - MSU053	*Ips cribricollis*	Mex, South of Amecameca	19.016	−98.741	11 v 2004	*Trich. tegucigalpae*	JN992201	JN992274
119 - MSU055	*Ips cribricollis*	Mex, Landa de Matamoros	21.263	−99.177	14 v 2004	*Trich. australis*	–	–
120 - MSU056	*Ips nitidus*	China, Sichuan			9 vii 2004	*Nent. eulaelaptis*	JN992135	JN992251
121 - MSU057	*Ips cribricollis*	Mex, Jalisco, nr. Ciudad Guzman			9 ii 2006	*Trich.* n.sp. 13	JN992198	–
122 - MSU060	*Ips pilifrons*	USA, CO, White River N.F. Lost Lake			30 vi 2005	*Trich. polytrichasimilis*	–	–
123 - MSU066	*Ips calligraphus*	USA, FL, Naples, Collier	26.157	−81.660	iii - iv 2007	*Trich. australis*	–	–
124 - MSU067	*Ips hoppingi*	USA, TX, McDonald Observatory			12 iv 2002	*Trich. californica*	JN992156	–
125 - MSU069	*Ips montanus*	USA, WA, Hwy 410, nr. Chinook Pass			11 v 2001	*Trich. polytrichasimilis*	–	–
126 - MSU071	*Ips pini*	USA, AK, Douglas is. nr. Juneau			4 v 2001	*Trich. idahoensis*	JN992168	–
127 - MSU073	*Ips pini*	USA, CA, Lassen N.F. Polesprings Rd.			3 vii 2001	*Trich. idahoensis*	JN992169	–
128 - MSU079	*Ips plastographus*	USA, CA,			v 2001	*Trich.* n.sp. 11	JN992197	JN992272
129 - MSU084	*Ips paraconfusus*	USA, CA, Mt. Diablo S.P. Contra Costa			10 vi 2001	*Trich.* n.sp. 7	–	–
130 - MSU085	*Ips lecontei*	USA, AZ, Coronado N.F. Ladybug Peak			18 vii 2001	*Trich. australis*	JN992148	–
131 - MSU086	*Ips cembrae*	Switzerland			v 2002	*Trich. polytricha*	JN992193	JN992270
132 - MSU090	*Ips montanus*	USA, CA, El Dorado, Hwy 50 nr. Meyer			13 vi 2001	*Trich. polytricha*	JN992194	–
133 - MSU091	*Pityogenes chalcographus*	Norway			v 2002	*Trich.* n.sp. 5	JN992180	JN992267
134 - MSU094	*Ips confusus*	USA, NV, Mt. Charleston Recreation	36.16	−115.32	27 vi 2003	*Trich. californica*	JN992157	–
135 - MSU099	*Ips confusus*	USA, UT, nr. Baker Dam	37.23	−113.39	28 vi 2003	*Trich. californica*	JN992158	–
136 - MSU104	*Ips confusus*	USA, AZ, Kaibab N.F. Hwy 389	36.51	−112.16	30 vi 2003	*Trich. californica*	JN992159	–
137 - MSU108	*Ips confusus*	USA, AZ, Kaibab N.F. nr. Flagstaff	35.24	−111.35	2 vii 2003	*Trich. californica*	JN992160	–
138 - MSU111	*Ips confusus*	USA, NM, Carson N.F. nr. Los Pinons	36.25	−106.01	9 vi 2003	*Trich. californica*	JN992161	JN992257
139 - MSU114	*Ips confusus*	USA, NM, Santa Fe			17 vi 2003	*Trich. californica*	JN992162	–
140 - MSU119	*Ips confusus*	USA, NV, Risue Canyon			4 vi 2003	*Trich. californica*	JN992163	–
141 - MSU123	*Ips confusus*	USA, AZ, Coconino, nr. Red Mt.	35.31	−111.5	vi 2003	*Trich. californica*	JN992164	–
142 - MSU124	*Ips confusus*	USA, CO, F.R. 504	37.669	−108.70	9 viii 2004	*Trich. californica*	JN992165	–
143 - MSU125	*Ips perturbatus*	Can, ON, Marlborough Forest			19 v 1995	*Trich. australis*	–	–
144 - MSU127	*Pseudips mexicanus*	USA, CA, San Francisco			20 viii 1995	*Trich.* n.sp. 9	JN992182	–
145 - MSU131	*Ips emarginatus*	USA, CA, Lassen, Black Mt.			7 vii 1995	*Trich. polytrichasimilis*	–	–
146 - MSU132	*Ips calligraphus*	USA, NY, Smithtown			11 ix 1994	*Trich. australis*	–	–
147 - MSU133	*Ips pini*	USA, NY			18 x 1995	*Trich. australis*	–	–
148 - MSU137	*Ips paraconfusus*	USA, CA, Mt. Diablo			3 ix 1995	*Trich.* n.sp. 7	JN992199	–
149 - MSU139	*Ips woodi*	USA, AZ, Coronado N.F. Hospital Flat			4 ix 1996	*Trich. polytricha*	JN992195	–
150 - MSU143	*Dendroctonus valens*	USA, PA, 225 Yeager Rd. Woodland	41.049	−78.349	30 iv 2009	*Uroob. americana*	JN992208	–
151 - MSU144	*Ips woodi*	USA, AZ, Apache N.F. Hannagan Meadow			1 ix 1996	*Trich. polytricha*	JN992196	–
152 - MSU147	*Ips pilifrons*	USA, AZ, Apache N.F. Hannagan Meadow			31 viii 1996	*Trich. australis*	JN992149	–
153 - MSU148	*Ips cribricollis*	USA, NM, Otero			v 1994	*Trich. australis*	JN992150	–
154 - MSU150	*Ips hunteri*	USA, AZ, Apache N.F. Hannagan Meadow				*Trich. australis*	JN992151	–
155 - MSU152	*Pseudips mexicanus*	USA, CA, Albion River Rd. nr. Rt. 1			23 iii 1996	*Trich.* n.sp. 9	JN992183	JN992268
156 - MSU154	*Ips emarginatus*	USA, CA, El Dorado N.F. Ice House Res.			6 ix 1997	*Uroob. orri*	JN992245	–
157 - MSU155	*Dendroctonus valens*	USA, CA, University of California Berkeley			14 x 1996	*Uroob. vinicolora*	JN992249	–
158 - MSU157	*Ips cribricollis*	USA, NM, Cloudcroft			11 v 1994	*Trich. australis*	JN992152	–
159 - MSU162	*Ips bonanseai*	Mex, Nuevo Leon			xii 1993	*Trich. tegucigalpae*	–	–
160 - MSU163	*Ips hoppingi*	Mex, Nuevo Leon	24.505	−99.985	25 x 1993	*Trich. californica*	–	–
161 - MSU167	*Ips plastographus*	USA, CA, Santa Cruz			13 x 1993	*Uroob. orri*	JN992246	–
162 - MSU168	*Ips pini*	USA, RI, Lincoln S.P.			19 vii 1997	*Trich. australis*	JN992153	–
163 - MSU173	*Ips emarginatus*	USA, CA, Lassen, Bogard Bultes			6 xii 1996	*Uroob. orri*	JN992247	–
164 - MSU174	*Ips cembrae*	Germany, Dresden			28 v 1986	*Trich. polytricha*	–	–
165 - MSU179	*Gnathotrichus materiarius*	USA, MI, Mt. Pleasant			28 v 1998	*Trich. parisiana*	JN992189	–
166 - MSU180	*Camptocerus auricomis*	Panama			4 ix 2008	*Trich.* n.sp. 6	–	–
167 - MSU185	*Corthylus* sp.	Panama	8.862	−82.743	26 viii 2008	*Trich.* n.sp. 1	JN992176	–
168 - MSU010	*Dendroctonus valens*	USA, PA, Keystone Rd.	40.739	−76.308	30 iv 2009	*Uroob. americana*	–	–
169 - MSU084	*Ips paraconfusus*	USA, CA, Mt. Diablo S.P. Contra Costa			10 vi 2001	*Trich.* n.sp. 7	JN992200	JN992273
170 - MSU123	*Ips confusus*	USA, AZ, Coconino, nr. Red Mt.	35.31	−111.5	vi 2003	*Trich. californica*	–	JN992258
171 - MSU143	*Dendroctonus valens*	USA, PA, 225 Yeager Rd. Woodland	41.049	−78.349	30 iv 2009	*Uroob. americana*	JN992209	JN992276
172 - MSU148	*Ips cribricollis*	USA, NM, Otero			v 1994	*Trich. australis*	JN992154	JN992256
173 - MSU154	*Ips emarginatus*	USA, CA, El Dorado N.F. Ice House Res.			6 ix 1997	*Uroob. orri*	–	–
174 - MSU185	*Corthylus* sp.	Panama	8.862	−82.743	26 viii 2008	*Trich.* n.sp. 1	JN992177	JN992264
175 - MSU025	*Hylurgops* sp.	Mex, South of Amecameca	19.016	−98.741	11 v 2004	*Uroob. vinicolora*	JN992250	JN992281
176 - MSU049	*Ips pilifrons utahensis*	USA, CO, San Isabel N.F. Monarch Pass	38.31	−106.19	9 vi 2003	*Trich. polytricha*	–	JN992271
177 - MSU179	*Gnathotrichus materiarius*	USA, MI, Mt. Pleasant			28 v 1998	*Trich. parisiana*	JN992190	JN992269
2 - WKN084	*Nicrophorus sayi*	Can, QC, Pont-Rouge	46.806	−71.679	05 vi 2009	*Uroob.* sp. 2	JN992096	–
7 - WKN165	*Nicrophorus orbicollis*	Can, ON, Carbine Rd.	45.330	−76.371	23 vii 2009	*Uroob.* sp. 1	JN992074	JQ316464
8 - WKN184	*Nicrophorus vespilloides*	Germany, Mooswald Forest, nr. Freiburg	48.0	7.85	vi 2009	*Uroob.* sp. 3	JN992102	JQ316465
21 - WKN165	*Nicrophorus orbicollis*	Can, ON, Carbine Rd.	45.330	−76.371	23 vii 2009	*Uroob.* sp. 1	JN992075	–
30 - WKN090	*Nicrophorus nepalensis*	Taiwan, nr. Meifeng, 5 km Sungkang	24.088	121.171	02 v 2007	*Uroob.* sp. 4	JN992103	–
65 - WKN350	*Nicrophorus sayi*	Can, NS, Portobello	44.75	−63.6	2009	*Uroob.* sp. 2	JN992097	–

Pairwise distances were calculated using neighbour-joining (NJ) analyses with the Kimura-2-parameter (K2P) model in PAUP* v4.0b10 [34]. Phylogenetic reconstructions of COI, 28S, and concatenated datasets were performed using Bayesian inference (BI) in MrBayes v3.1.2 [35], [36], and parsimony analyses in TNT v1.1 [37]. Gaps were treated as missing since gaps scored as a fifth state produced the same topology as that observed for gaps as missing for each of the analytical approaches. Analyses of the COI dataset excluding the third codon positions produced poorly supported reconstructions with similar topology to the analyses including the third codon position; hence analyses were performed including the 3^rd^ codon.

MrModeltest v2.3 [38] was used to determine the best-fit model of molecular evolution for each gene, which was determined to be GTR+I+G. Bayesian analysis was performed in MrBayes with a Markov Chain Monte Carlo (MCMC) method, two independent runs, with nucmodel = 4by4, N_st_ = 6, rates = invgamma, samplefreq = 1000, four chains = one cold and three heated. The COI dataset ran for 20 million generations, and the 28S and concatenated datasets ran for 10 million generations with a burn-in of 1000. In Mesquite, the remaining trees, excluding the burn-in, were used to generate a majority-rule consensus tree displaying the posterior probability supports for each node. Bayesian analyses were performed using the on-line Computational Biology Service Unit at Cornell University, and at the Cyberinfrastructure for Phylogenetic Research (CIPRES) portal [39].

Parsimony analysis was performed using a heuristic search with tree bisection-reconnection (TBR) branch swapping and 1000 random addition sequence replicates, all characters were treated as unordered, equal weighting, and gaps were treated as missing. Multiple trees were obtained and these were presented in a semistrict consensus tree. Node support was assessed in TNT, using jackknife resampling with 36% of characters removed and 1000 replicates, Bremer supports and partitioned Bremer supports (PBS) were also determined using TNT. Node support for the parsimony analysis of the COI and concatenated datasets were mapped onto the corresponding Bayesian phylogenies.

### Morphological Analysis

To assess intraspecific morphological divergence of mites used in the molecular analyses, slide-mounted specimens were examined using a Leica DM5500B compound microscope, and 15 and 14 characters (for Trematuridae and Urodinychidae, respectively) were measured using Leica Application Suite, Live and Interactive Measurements Modules v3.5. Characters from different body regions were selected based on their relative ease of measurement and prominence, as well as previously observed variation across specimens. The 15 characters measured for trematurid species were: maximal length and width of the dorsal shield and ventrianal shield; sternal shield (SS) median length; SS width at five levels (from anterior to posterior): maximal width of the SS anterior margin, maximum width of the two expansions at level with coxae II–III and coxae III–IV, minimum width of the posterior constriction level with coxa IV, and width of the SS posterior margin; length of tarsus I; and the length of the following setae: opisthogastric setae *V8* and *V4* [25] (*JV4* and paranal, sensu [40]), the proximoventral setae of femur I, and the longest of anterodorsal setae in the sensory pit of tarsus I. The same characters were measured for Urodinychidae (*Uroobovella*) species, except that seta *V4* and proximoventral setae of femur I were not measured, but the length of dorsal seta *j1* was instead. Morphological divergence was visualized by generating an ordination based on semistrong hybrid multidimensional scaling (SSH MDS) with PATN v2.27 [41]. The ordination was based on a Bray-Curtis distance matrix between mite specimens created using morphometric data standardized for body size to eliminate bias linked to body size, and transformed ((value – minimum)/range) to balance the weight of all measured characters. The ordination was generated based on 1000 iterations and 1000 random starts. Significant differences among groups detected in a given ordination were tested using ANOSIM (analysis of similarity), with 1000 iterations.

To ensure that specimens that underwent DNA extraction could be studied morphologically without any bias, the effect of DNA extraction was tested by comparing the morphology of specimens that underwent DNA extraction with specimens of the same species, and from that same host individual, that did not undergo extraction. Thirteen of the aforementioned morphological characters (standardized for body size) were examined for specimens of two species (*Uroobovella orri*, *Trichouropoda californica*) using Wilcoxon signed rank tests performed in SPSS v17 (SPSS Inc., Chicago, United States of America). No significant differences in morphology were observed between *U. orri* mites that underwent DNA extraction versus mites that did not undergo extraction, based on 13 characters and 15 pairwise comparisons (each pair consisting of two mites from the same host individuals; *P* = 0.078–0.995). DNA extraction had no significant effect on the morphology of *T. californica* specimens either (*P* = 0.139–0.799; 13 characters, 10 pairwise comparisons), except for two characters: median length and width of the sternal shield (*P* = 0.037, *P* = 0.009). The variation of these characters was most likely an artefact of slide mounting following DNA extraction, in that extraction weakens sclerotized tissue, which may have encouraged shields to fracture. Slide-mounted *T. californica* specimens that underwent DNA extraction had small fractures on either side of the sternal shield just posterior to the midpoint, and this may have increased sternal shield medial length and width measured relative to that of mites that did not undergo DNA extraction. With the exception of these two characters, DNA extraction did not significantly alter mite morphology, and as a result specimens that underwent extraction can be compared morphologically without any incurred bias.

## Results

**Figure 2:**
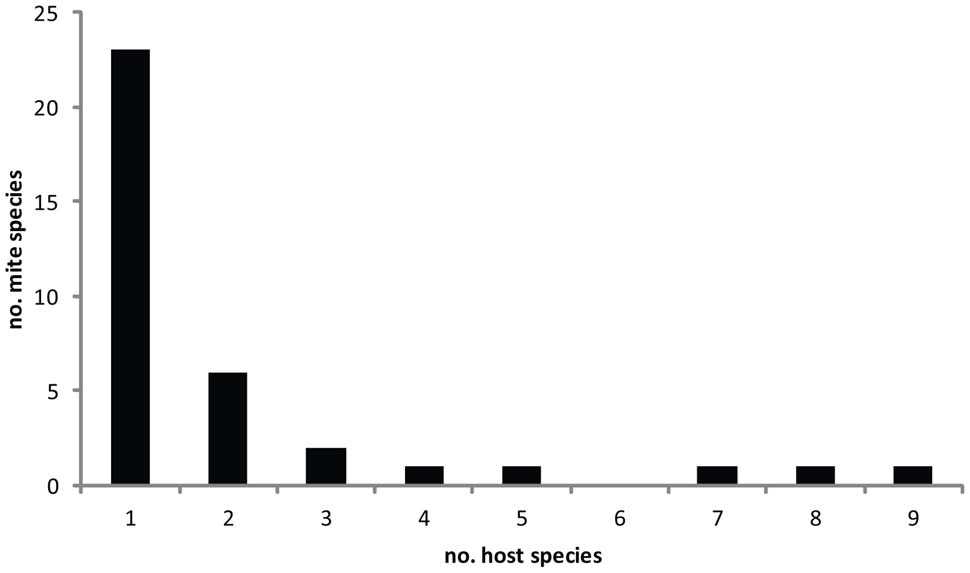
Distribution of the breadth of host range of uropodoid mites. Uropodoids collected from 51 species of bark beetles from 11 countries, showing the number of total mite species and the number of scolytine species used by each mite species. Note that these observed host ranges are based on opportunistic sampling from various regions; therefore, the true host ranges are possibly much broader.

A total of 36 species of uropodoids (from three genera and two families) were found on 51 scolytine species (from 20 genera and 10 tribes), which were collected across 11 countries (Table 2). Of these 36 mite species, 13 are undescribed. The majority of the 36 species were collected from only one (64%) or two (17%) host species; fewer species were collected from three to nine host species (19%) (Fig. 2, Table 2). Most (76%) of the host associations observed in this study represent new records, and 19 of the 23 described species collected in this study had new host records (Table 3). There was little overlap in bark beetle hosts between this study and the literature for many of the common uropodoid species (e.g. *T. australis*, *T. polytricha*, and *U. orri*, each with only 1–3 host species shared; Table 3). The host records of many of the described species collected in this study are novel, when compared with published host records (Table 3). Most bark beetle species were associated with only one or two mite species; four host species had three mite species, and one host species (*Polygraphus rufipennis*) was associated with four mite species (Table 2).

**Table 3:** Comparing observed host records (this study) with published records (publ.) for described mite species collected from scolytines and other families of wood-boring beetles^1^ (*number of host spp. shared).

Mite species	No. host spp/genera		Published host species (°spp. shared with present study)	Regions^2^	References
	This study	Publ.			
*Nenteria chiapasa*	1	0	pine duff (needle litter)	Mexico	54
*N. eulaelaptis*	1	0	no host or habitat provided	Hungary, Mongolia	25, 54
*N. moseri*	1	1	*Dendrocontus frontalis*	Guatemala	55
*Trichouropoda alascae*	1	2*/1	*Dendroctonus obesus, D. rufipennis°*	AK	28,56
*T. australis*	8/1	12***/3	*Dendroctonus brevicomis, D. frontalis, D. ponderosae, D. terebrans,* *D. simplex, Ips avulsus, I. bonanseai, I. calligraphus°, I. confusus,* *I. grandicollis°, I. pini°;* CER: *Neacanthosinus obsoletus*	AZ, LA, MS, TX	9,57,58
*T. bipilis*	1	1	*Scolytus pygmaeus*	Austria	29
*T. californica*	2/1	1*	*Ips confusus°*	CA	59
*T. fallax*	3/2	5*/3	*Dendroctonus adjunctus, Hylastes ater, H. cunicularius,* *H. interstitialis, Hylurgops pinifex°*	LA; Siberia; Belgium	29,57
*T. hirsuta*	4/4	15/7	*Dendroctonus approximatus, D. brevicomis, D. frontalis, D. valens,* *Gnathotrichus materiarius, Ips avulsus, I. calligraphus, I. grandicollis, I. pini,* *Trypodendron scabricollis;* CER: *Monochamus carolinensis, M. scutellatus,* *M. titillator, Neacanthosinus obsoletus, Xyloterus sagittatus*	AB, ON; AZ, LA, MS, TX	9,27,57,58,60
*T. idahoensis*	1	1*	*Ips pini°*	ID	27
*T. lamellosa*	2/2	10*/6	*Dendroctonus pseudotsugae, Dryocoetes confusus, Ips avulsus,* *I. calligraphus, I. grandicollis°;* CER: *Monochamus carolinensis, M. scutellatus,* *M. titillator, Neacanthosinus obsoletus, Xyloterus sagittatus*	AB, ON; AZ, LA, MS	9,14,57,58,60
*T. moseri*	2/2	1	*Dendroctonus simplex*	AB	25
*T. parisiana*	3/3	2/1	*Ips sexdentatus, I. typographus*	France	28
*T. perissopos*	1	1	CUR: *Perissops sobrinus*	Poland	27
*T. polygraphi*	1	1	*Polygraphus minor*	India	29
*T. polytricha*	7/1	7*/4	*Dryocoetes autographus, Hylurgops palliatus, Ips amitinus, I. cembrae°,* *I. hauseri, I. typographus, Pityogenes chalcographus*	Austria, Germany, Poland, Turkey	29,61
*T. polytrichasimilis*	3/1	1	*Ips sexdentatus*; under bark of *Pinus pinaster*	France, Portugal	25,62
*T. tegucigalpae*	2/1	3**/2	*Dendroctonus frontalis, Ips bonanseai°, I. cribricollis°*	Honduras, Mexico	27
*Uroobovella americana*	1	7*/3	*Dendroctonus pseudotsugae, D. terebrans, D. valens°, Gnathotrichus* *materiarius, Ips avulsus, I. calligraphus, I. grandicollis*	AZ, LA	9,57
*U. australiensis*	1	1	CER: *Pelargoderus arouensis*	Australia	63
*U. dryocoetes*	5/4	3*/3	*Dryocoetes autographus°, Hylastes cunicularius, Ips sexdentatus*	Austria	29
*U. orri*	9/6	11**/4	*Dendroctonus brevicomis, D. frontalis, D. obesus, D. pseudotsugae,* *D. valens, Dryocoetes confusus, Gnathotrichus materiarius°, Ips avulsus,* *I. calligraphus, I. grandicollis°, I. pini.*	AZ, LA, MS, TX	9,57
*U. vinicolora*	2/2	1	*Ips typographus*	Germany	61

1 CER  =  Cerambycidae, CUR  =  Curculionidae.

2 Provinces and states of Canada and USA follow accepted abbreviations.

Amplification of COI was attempted with 176 deutonymphal mites, from which only 116 (representing 29 species and three genera) from nine countries and 74 sites yielded sequence data (Table 2). COI was amplified from 122 specimens (116 ingroup and six outgroup specimens), with 608 characters in total, 328 constant, 19 parsimony-uninformative, and 261 parsimony-informative. Mean base pair frequencies (A: 0.294, C: 0.187, G: 0.153, T: 0.366) were found to be heterogeneous across all specimens (χ^2^ = 504.83, *P*<0.0001). The 28S D2–D4 region was used to assess the branching patterns observed in the COI reconstructions and to further test species boundaries. Partial 28S was amplified from 31 mites from 25 species (three genera) collected across nine countries and 26 sites, as well as from two outgroup specimens (Table 2), with 1069 characters in total, 446 constant, 114 parsimony-uninformative, and 509 parsimony-informative. Mean base pair frequencies (A: 0.239, C: 0.199, G: 0.283, T: 0.279) were found to be homogeneous across all specimens (χ^2^ = 92.12, *P* = 0.59). In each reconstruction, each specimen is labeled with a unique number, followed by the host species and abbreviated state, province or country (Table 2).

### Pairwise Divergence

NJ analysis (K2P) of COI was performed on 122 mite specimens including 116 ingroup specimens (29 spp. total: 21 *Trichouropoda*, 2 *Nenteria*, and 6 *Uroobovella* spp.) and six outgroup specimens (four spp.). Average COI intraspecific pairwise distance was lowest among *Trichouropoda* species (1.5%±1.8) and slightly higher among *Uroobovella* species (1.9%±2.9) (Table 4). The maximum intraspecific divergence was high for both genera, with a maximum of 10.4% for *T. polytricha* and 12.5% for *U. orri*, both of which were between new and old world specimens (Table 4). Mean interspecific divergence within each genus was relatively high for all three genera (16.7–17.3%), and typically greater than intraspecific divergence (Table 4). The maximum divergence between *Trichouropoda* species was between *T. hirsuta* and *T. moseri* (23.4%), and the minimum was between *T.* n.sp. 11 and *T. idahoensis* (0.5%). The maximum for *Uroobovella* was between *U. americana* and *U. orri* (20.8%), and the minimum was between *U. americana* and *U. vinicolora* (8.4%) (Table 4). Average intergeneric divergence was high (18.6–21.5%), with the maximum divergence between *T. hirsuta* and *U. australiensis* (28.1%) (Table 4).

**Table 4:** Intra- and interspecific nucleotide divergence (%) ±standard deviation (range) of COI and 28S amplified from uropodoid mites associated with bark beetles.

	COI	28S
	mean (range)	mean (range)
**Intraspecific**		
* Trichouropoda*	1.5±1.8 (0–10.4)	0.3±0.2 (0.1–0.5)
* Nenteria* 1	–	–
* Uroobovella*	1.9±2.9 (0–12.5)	0.0±0.0 (0)
**Interspecific**		
* Trichouropoda*	16.7±2.9 (0.5–23.4)	7.1±5.0 (0–16.6)
* Nenteria*	16.9±0.0 (16.9)	10.0±0.0 (10.0)
* Uroobovella*	17.3±2.7 (8.4–20.8)	32.7±15.9 (1.5–42.5)
**Intergeneric**		
* Trich – Nent*	18.6±1.2 (16.3–23.2)	16.0±1.1 (13.8–20.0)
* Trich – Uroob*	21.3±1.4 (17.7–28.1)	34.9±3.9 (28.5–41.6)
* Nent – Uroob*	21.5±1.3 (18.6–23.6)	34.5±3.7 (29.0–41.1)

1 *Nenteria* was represented by only 2 species, and each by a single individual.

NJ analysis of 28S was performed on 33 mite specimens including 31 ingroup specimens (25 spp. total: 18 *Trichouropoda*, 2 *Nenteria*, and 5 *Uroobovella* spp.), and two outgroup species. Average 28S intraspecific pairwise distance was highest among *Trichouropoda* species (0.3%±0.2), and lowest among *Uroobovella* species (0%±0) (Table 4). The maximum intraspecific divergence was relatively low for *Trichouropoda* with a maximum of 0.5% for *T. californica*, and low for *Uroobovella* with a maximum of 0% for *U.* n.sp. 6 and *U. americana* (Table 4). Mean interspecific divergence within each genus was moderate to very high (7.1–32.7%), and clearly higher than intraspecific divergence (Table 4). The maximum between *Trichouropoda* species was between *T. hirsuta* and *T.* n.sp. 11 (16.6%), and the minimum was between *T. lamellosa* and *T.* n.sp. 10 (0%) (Table 4). The maximum for *Uroobovella* species was between *U. dryocoetes* and *U. orri* (42.5%), and the minimum was between *U. vinicolora* and *U. americana* (1.5%) (Table 4). Average intergeneric divergence was high (16.0–34.9%), with the maximum pairwise distance between *Trichouropoda lamellosa* and *Uroobovella dryocoetes* (41.6%) (Table 4).

### Bayesian Inference

**Figure 3:**
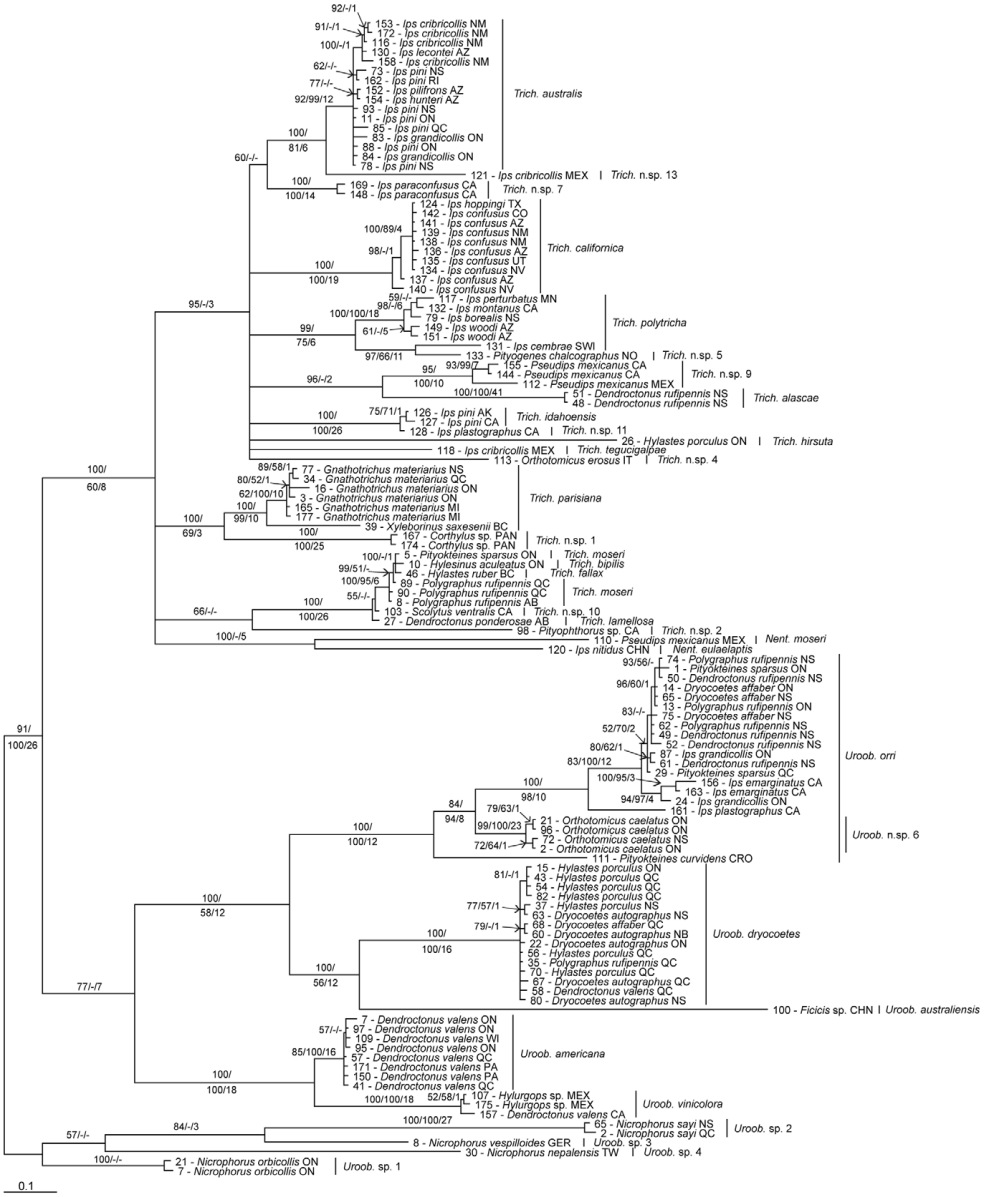
Bayesian majority rule consensus tree based on COI from bark beetle associated uropodoids. Majority rule consensus tree of 38002 trees generated by Bayesian MCMC analysis (20 million generations) of 608 bp fragment of COI from 122 uropodoid specimens, 116 ingroup specimens representing 29 species, and six outgroup specimens representing four species (TL = 2021, CI = 0.2459, RI = 0.8277) (*Uroob*. =  *Uroobovella*, *Trich*. =  *Trichouropoda*, *Nent*. =  *Nenteria*). Posterior probability >50%/jackknife support >50%/Bremer support (JKS and BS from parsimony analysis).

**Figure 4:**
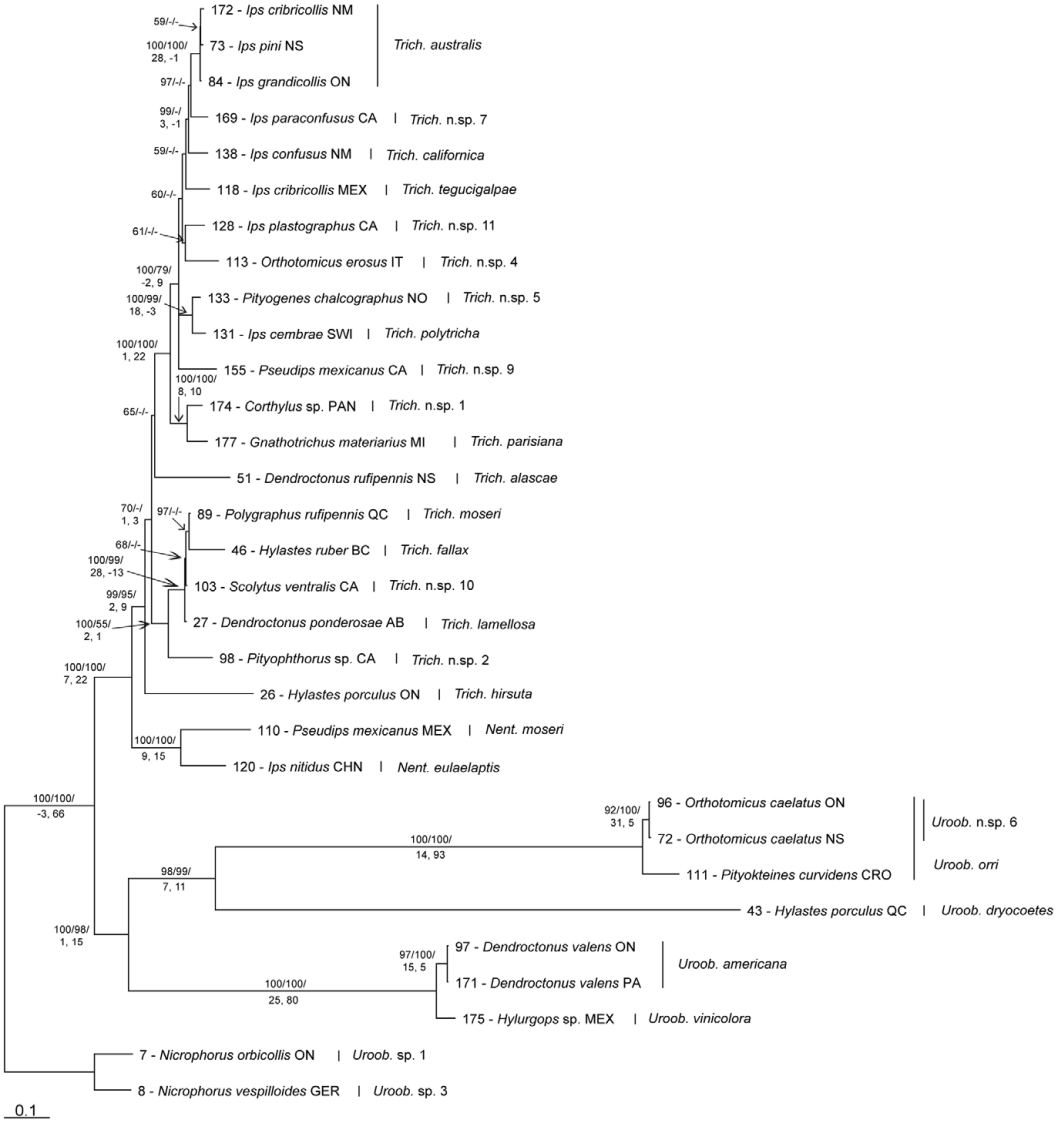
Bayesian majority rule consensus tree based on COI and 28S from bark beetle associated uropodoids. Majority rule consensus tree of 18002 trees generated by Bayesian MCMC analysis (10 million generations) of concatenated dataset of 608 bp fragment of COI and 1069 bp fragment of 28S from 31 specimens, 29 ingroup specimens representing 25 species, and two outgroup species (TL = 2947, CI = 0.4964, RI = 0.6746) (*Uroob*. =  *Uroobovella*, *Trich*. =  *Trichouropoda*, *Nent*. =  *Nenteria*). Posterior probability >50%/jackknife support >50%/partitioned Bremer support (COI, 28S) (JKS and PBS from parsimony analysis).

BI of COI was performed for 20 million generations, producing 38002 trees (after burn-in) which were summarized in a majority rule consensus tree (TL = 2021, CI = 0.2459, RI = 0.8277) (Fig. 3). The BI consensus tree was well supported, with most nodes having moderate to high posterior probabilities, with 26 nodes having 100% support, eight of which are basal nodes to ingroup species (Fig. 3). Some species, such as *T. australis*, *T. californica*, *U. orri*, *U. dryocoetes*, and *U. americana*, had multiple unresolved nodes collapsing into intraspecific polytomies. BI of 28S was performed for 10 million generations, producing 18002 trees (after burn-in) that were summarized in a majority rule consensus tree (TL = 1465, CI = 0.6881, RI = 0.8204) (tree not shown). The consensus tree was well supported: 12 nodes had 100% support, one of which was the node to the ingroup. BI of the concatenated dataset was performed for 10 million generations, producing 18002 trees (after burn-in) which were summarized in a majority rule consensus tree (TL = 2947, CI = 0.4964, RI = 0.6746) (Fig. 4). The total evidence consensus tree was well supported: 13 nodes had 100% support, including the basal node to the ingroup (Fig. 4).

### Parsimony

The parsimony heuristic analysis of COI resulted in 34 most parsimonious trees (TL = 1928, CI = 0.2578, RI = 0.8383) presented in a semistrict consensus tree (tree not shown). Many nodes had moderate to high JKS which were mapped onto the Bayesian analysis of COI (Fig. 3), 18 nodes had 100% jackknife support (JKS). Many nodes had poor Bremer support, with 24 nodes with moderate to strong support (≥10), as shown in the Bayesian phylogeny (Fig. 3). Nine of the nodes with 100% JKS and strong Bremer support are basal nodes to ingroup species. Similar to the BI, *T. australis*, *T. californica*, *U. orri*, *U. dryocoetes*, and *U. americana* had multiple unresolved nodes collapsing into intraspecific polytomies. The heuristic analysis of 28S produced 14 most parsimonious trees (TL = 1462, CI = 0.6895, RI = 0.8216) presented in a semistrict consensus tree (tree not shown). Most nodes had moderate to strong Bremer support and nearly every node had JKS, with 12 nodes having 100% JKS, one of which was the basal node to the ingroup. Multiple *Trichouropoda* species showed little interspecific divergence resulting in a large polytomy. The parsimony analysis of the concatenated dataset resulted in three most parsimonious trees (TL = 2924, CI = 0.5003, RI = 0.6797) presented in a semistrict consensus tree (tree not shown). Most nodes had moderate to strong JKS, with 10 nodes having 100% JKS, including the basal node to the ingroup and to the Trematuridae, and many nodes had moderate to strong PBS, as shown in the Bayesian analysis (Fig. 4).

### Summary of Molecular Reconstructions

The parsimony and Bayesian analyses of COI, 28S and concatenated datasets yielded similar results. All COI analyses suggested that each trematurid (*Trichouropoda* and *Nenteria*) species was monophyletic, with the exception of *T. moseri* and *T. polytricha*. *Trichouropoda moseri* collected from *Pityokteines sparsus* consistently grouped separately from those collected from *Polygraphus rufipennis*. *Trichouropoda polytricha* collected from *Ips cembrae* from Switzerland was consistently shown to be more closely related to *T.* n.sp. 5 from Norway than to other North American *T. polytricha* specimens.

Overall, the relationships between trematurid species were poorly resolved using 28S, with slightly better resolution in the concatenated dataset, and the best resolution using COI alone. The D2–D4 region of 28S was not effective for examining the relationships between some closely related *Trichouropoda* species. The 28S and COI analyses were not entirely congruent. In all 28S reconstructions, *T. hirsuta* was basal to all other species in the genus, whereas *T.* n.sp. 2 was the basal species in COI reconstructions. COI and 28S also disagreed on the placement of *T. fallax* and *T. alascae*. COI provided more insight into the relationships between trematurid species than 28S. The concatenated dataset produced well-supported trees, which were more resolved than those based on 28S alone. The placement of a few *Trichouropoda* species differed between the 28S and concatenated reconstructions, reflecting the differences in trematurid species relationships independently inferred from COI versus 28S.

Across all reconstructions the monophyly of all *Uroobovella* species were well supported and the relationships between *Uroobovella* species were consistent across all analyses. In particular, *U. orri, U.* n.sp. 6, *U. dryocoetes* and *U. australiensis* appear to be most closely related to each other, whereas *U. americana* and *U. vinicolora* are most closely related to each other. Across all COI analyses there was a small well-supported clade grouping *U. orri* specimens from *Orthotomicus caelatus* beetles, which has been labeled as *U.* n.sp. 6.

### Morphological Analysis

**Figure 5:**
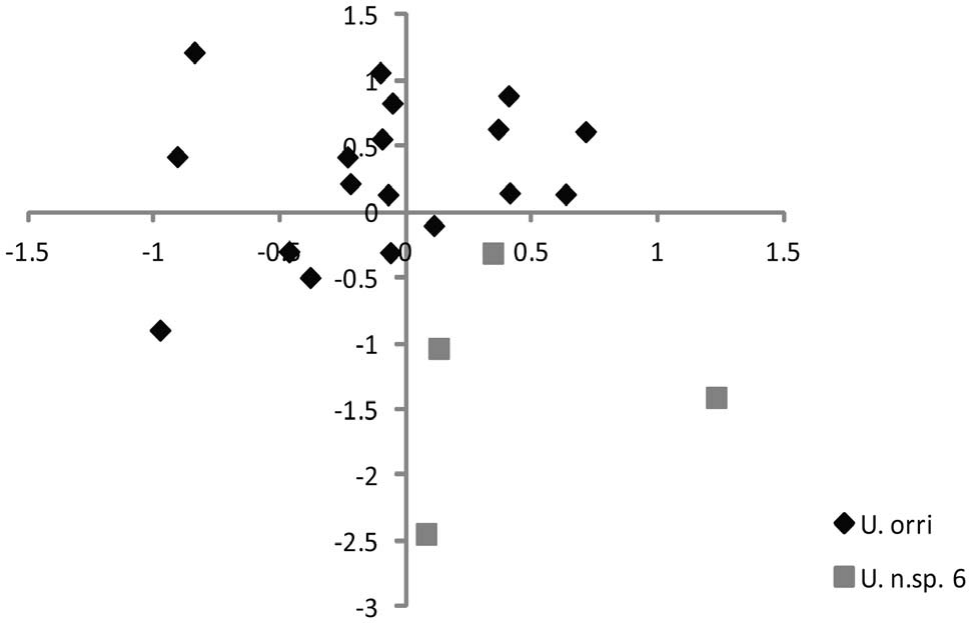
SSH MDS ordination showing morphological dissimilarity among *Uroobovella* species. Ordination with Bray-Curtis distance performed on measurements ((value – min)/range transformed) of 14 morphological characters from 22 uropodoids representing *U. orri* and *U.* n.sp. 6 (stress = 0.1571).

To test whether host generalists displayed cryptic morphological diversity, the level of ‘intraspecific’ morphological divergence was assessed in five species with broad host ranges (*T. australis*, *T. parisiana*, *T. polytricha*, *U. orri*, *U. dryocoetes*), and two species with relatively narrow host ranges (*T. californica* and *U. americana*). *Uroobovella orri* was the only species of the seven examined that showed prominent morphological variation, with two apparent groupings in the ordination: mites from *Orthotomicus caelatus*, labelled as *U.* n.sp. 6, and mites from hosts (8 host spp.) other than *O. caelatus* (Fig. 5). The SSH MDS ordination (stress = 0.1571) (Fig. 5) and ANOSIM based on 14 morphological characters measured from 22 *U. orri* specimens indicate that *U. orri* and *U.* n.sp. 6 are significantly distinct morphologically (*P* = 0.01). Subsequently, slide-mounted specimens were examined closely for variation in discrete morphological characters that could be used to distinguish *U. orri* and *U.* n.sp. 6, but this investigation revealed no distinct character states. Mean COI divergence among *U.* n.sp. 6 specimens was low (0.5% ±0.31), where as the mean divergence between *U.* n.sp. 6 and other *U. orri* specimens from North America was 20 times higher (10.5% ±0.4).

The remaining six generalist and two species with narrow host ranges displayed no significant intraspecific variation in morphometrics or discrete (qualitative) morphological characters; these species also showed low COI intraspecific divergence (<1%), with the exception of *T. polytricha* and *T. parisiana* with 4.6% (±3.8) and 2.8% (±2.7) divergence, respectively. The relatively high level of divergence among *T. polytricha* specimens was largely due to a single specimen from Switzerland; intraspecific divergence among North America specimens was 2% (±0.8).

## Discussion

This study indicates that both partial COI and 28S D2–D4 are suitable markers for distinguishing between closely related uropodoid species, with 17% average divergence among species for both markers. 28S appears to be a good marker for separating closely related *Uroobovella* species, but COI was far more effective at delineating between *Trichouropoda* species. Most morphologically defined species were well supported in the COI phylogeny, with the exception of *T. moseri* and *T. polytricha*. The congruence between morphological and molecular data emphasizes the fact that the best approach is an integrative approach [42], and that morphology-based taxonomy is still relevant and essential [43].

### Host Specificity and Cryptic Species

A total of 36 species of uropodoids, including 13 undescribed species, were collected in this study, and these mites exhibited various levels of host specificity. The majority of mite species were collected from one (64%) or two (17%) host species, and seven species (19%) had three or more host species. However, the opportunistic sampling used in this study and the haphazard coverage of hosts and regions may incur a bias towards higher apparent host specificity. Considering published host records, it appears that strict host specificity may be the exception rather than the rule. The observed host associations in this study nearly doubled the number of host records for the described species studied (54% increase from 87 records to 134), and this highlights the lack of knowledge in this group. Considering that only a small proportion of the global bark beetle fauna has been examined for uropodoids, we suspect that many more new and/or cryptic species may be uncovered with further investigations.

Typically, when the species boundaries of symbiotic taxa are assessed using molecular techniques it is revealed that apparent generalists are actually complexes of cryptic specialists (e.g. [5], [7], [44]). To the contrary, in this study molecular and morphological analyses suggested that putative host generalists do not represent complexes of cryptic species with narrower host ranges, but that they are truly single species with a broad host range, with the exception of one species (*U. orri*). It is possible that some of these apparent generalists comprise rare specialists that remain to be collected, or that additional markers may uncover cryptic specialists, but it is also possible that these species are truly generalists.

*Uroobovella orri* was the only host generalist that appears to represent at least two distinct species in North America, including a widespread generalist associated with at least eight species and six genera of hosts, and a specialist (*U.* n.sp. 6) associated with *Orthotomicus caelatus* (based on COI data). Interestingly, *O. caelatus* is a host-tree generalist and attacks many species of *Pinus*, *Picea* and *Larix* throughout its range [8]. In addition, the single specimen of *U. orri* found on *Pityokteines curvidens* (another conifer generalist) from Croatia may also represent a distinct cryptic species, based upon the level of COI divergence from other *U. orri* specimens (11.5% ±0.7). Considering that *U. orri* has been collected from many other bark beetle species that were not included in this study, it is possible that we have only begun to scratch the surface of a diverse complex of cryptic species.

In all COI reconstructions both *T. moseri* and *T. polytricha* were paraphyletic, and this may suggest that these two species represent multiple cryptic species associated with different hosts. *Trichouropoda moseri* collected from *Pityokteines sparsus* (Ipini) and *Polygraphus rufipennis* (Polygraphini) were paraphyletic, and these may represent two cryptic host-specific species rather than a single host generalist; however, no morphometric differences were found, and average COI divergence among *T. moseri* specimens was very low (0.4% ±0.2). *Trichouropoda polytricha* found on *Ips cembrae* from Switzerland was more closely related to *T.* n.sp. 5 from Norway (*Pityogenes chalcographus*) than to North American *T. polytricha*. Despite being apparently morphologically identical, it is possible that the North American and European *T. polytricha* represent two cryptic species. Alternatively, the paraphyly of *T. moseri* and *T. polytricha* may be a result of inadequate taxon sampling, or incomplete lineage sorting. More specimens and additional markers are needed to clarify the taxonomic boundaries of these two mites.

The host associations of the closely related uropodoids, *T. parisiana* and *T.* n.sp.1, are unique and likely warrant future investigations. *Trichouropoda parisiana* and *T.* n.sp. 1 were both associated with ambrosia beetles, an ecological grade of scolytine and platypodine curculionids that carry symbiotic fungi (in complex glandular mycangial structures) which is inoculated into host trees and cultivated as a food source [8]. *Trichouropoda parisiana* was collected from three distantly related ambrosia beetles, *Gnathotrichus materiarius* (Corthylini), *Xyleborinus saxesenii* (Xyleborini) and *Trypodendron retusum* (Xyloterini), which attack a broad range of unrelated host trees (*Pinus* and *Picea* spp.; numerous trees and shrubs; *Populus* spp., respectively) [8]. *Trichouropoda* n.sp. 1 is morphologically and genetically similar to *T. parisiana*, and it was only collected from *Corthylus* sp. (Corthylini), an ambrosia beetle associated with deciduous trees [8]. It is likely that a common ancestor of *T. parisiana* and *T.* n.sp. 1 was originally associated with ambrosia beetles, and that descendant populations tracked some aspect of the mycetophagous life history of their hosts. However, testing this hypothesis further will be difficult given that these two mites are associated with hosts that feed on unrelated host trees in different countries [8]. *Trichouropoda* n.sp. 6 and *T.* n.sp. 8 were also collected from ambrosia beetles, *Camptocerus auricomis* and *Monarthrum dentigerum* respectively; however, since neither species yielded COI or 28S data, the phylogenetic relationships between these species and *T. parisiana* and *T.* n.sp. 1 are not understood.

### Coevolution

The evolutionary history of associated symbionts may reflect a long-term coevolutionary relationship, or it may reflect a history of host switching and ecological tracking [45], [46]. Overall, the evolution of scolytine-associated uropodoids shows little evidence of coevolution with their hosts or tracking ecologically similar host species. Phylogenetically related bark beetles [47]–[49] did not necessarily share the same or closely related mite species, and ecologically related host species, which have similar host tree ranges, overlapping geographic ranges or similar phenologies [8], [50] were not necessarily associated with the same or closely related uropodoid species.

An obstacle to the study of coevolution between bark beetles and uropodoids is that phylogenetically related hosts are often ecologically similar (e.g. host tree species, habitat range, feeding ecology, and phenology; [8], [50]), making it difficult to discern the determinants of host associations. For example, *T. californica* is phoretic on two sister-species, *Ips hoppingi* and *I. confusus* [49]. However, *I. hoppingi* and *I. confusus* are peripatric and similar ecologically, both feeding on pinyon pine (*Pinus*) species [8], and therefore it is very difficult to pinpoint the causal factor(s) in the association of *T. californica* with these two host species. Additionally, the ecology of bark beetle associated uropodoids are poorly understood, which hampers any interpretations of the extent to which mites may be tracking ecologically similar hosts. Future investigations into the extent to which uropodoids may be coevolving with their bark beetle hosts will require much more extensive taxon sampling than that of this study, as well as a more complete and resolved phylogeny of associated mites and their scolytine hosts, and an improved understanding of the ecology of these mites.
